# Supplementary material for: Dynalign II: common secondary structure prediction for RNA homologs with domain insertions
Source: Nucleic Acids Res. 2014 Nov 21;42(22):13939–48. doi: 10.1093/nar/gku1172 (PMC4267632; doi:10.1093/nar/gku1172)
Supplement: SUPPLEMENTARY DATA [file supp_gku1172_nar-02021-z-2014-File012.zip › manual/RNA_class/html/class_thermodynamics-members.html]

RNAstructure Classes: Member List

- Main Page
- Classes
- Files

- Class List
- Class Hierarchy
- Class Members

# Thermodynamics Member List

This is the complete list of members for Thermodynamics, including all inherited members.

|  |  |  |
| --- | --- | --- |
| CopyThermodynamic(Thermodynamics \*thermo) | Thermodynamics |  |
| data | Thermodynamics | `[protected]` |
| energyread | Thermodynamics | `[protected]` |
| enthalpy | Thermodynamics | `[protected]` |
| GetDat(char \*loop, char \*stackf, char \*tstackh, char \*tstacki, char \*tloop, char \*miscloop, char \*danglef, char \*int22, char \*int21, char \*coax, char \*tstackcoax, char \*coaxstack, char \*tstack, char \*tstackm, char \*triloop, char \*int11, char \*hexaloop, char \*tstacki23, char \*tstacki1n, char \*datapath, bool isRNA, bool isEnthalpy=false) | Thermodynamics | `[protected]` |
| GetDatatable() | Thermodynamics |  |
| GetEnergyRead() | Thermodynamics |  |
| GetEnthalpyTable() | Thermodynamics |  |
| GetTemperature() | Thermodynamics |  |
| isrna | Thermodynamics |  |
| ReadThermodynamic(const char \*pathname=NULL) | Thermodynamics |  |
| SetTemperature(double temperature) | Thermodynamics |  |
| temp | Thermodynamics | `[protected]` |
| Thermodynamics(const bool ISRNA=true) | Thermodynamics |  |
| ~Thermodynamics() | Thermodynamics |  |

---

Generated on Tue Jul 30 10:52:29 2013 for RNAstructure Classes by 
 1.5.7.1
